# Bio-based protic salts as precursors for sustainable free-standing film electrodes

**DOI:** 10.1038/s41598-024-61553-x

**Published:** 2024-05-15

**Authors:** Alina Brzęczek-Szafran, Magdalena Gwóźdź, Bartłomiej Gaida, Maciej Krzywiecki, Mirosława Pawlyta, Agata Blacha-Grzechnik, Anna Kolanowska, Anna Chrobok, Dawid Janas

**Affiliations:** 1 Faculty of Chemistry, Silesian University of Technology, 44-100 Gliwice, Poland; 2 Department of Applied Physics, Institute of Physics CSE, Silesian University of Technology, 44-100 Gliwice, Poland; 3 Materials Research Laboratory, Faculty of Mechanical Engineering, Silesian University of Technology, 44-100 Gliwice, Poland

**Keywords:** Ionic liquids, Sustainability, Energy, Materials chemistry

## Abstract

Transforming amines with low boiling points and high volatilities into protic salts is a versatile strategy to utilize low molecular weight compounds as precursors for N-doped carbon structures in a straightforward carbonization procedure. Herein, conventional mineral acids commonly used for the synthesis of protic salts were replaced by bio-derived phytic acid, which, combined with various amines and amino acids, yielded partially or fully bio-derived protic salts. The biomass-based salts showed higher char-forming ability than their mineral acid-based analogs (up to 55.9% at 800°), simultaneously providing carbon materials with significant porosity (up to 1177 m^2^g^−1^) and a considerable level of N,P,O-doping. Here, we present the first comprehensive study on the correlation between the structure of the bio-derived protic precursors and the properties of derived carbon materials to guide future designs of biomass-derived precursors for the one-step synthesis of sustainable carbon materials. Additionally, we demonstrate how to improve the textural properties of the protic-salt-derived carbons (which suffer from high brittleness) by simply upgrading them into highly flexible nanocomposites using high-quality single-walled carbon nanotubes. Consequently, self-standing electrodes for the oxygen reduction reaction were created.

## Introduction

With the indispensable development of green energy technologies where oxygen evolution and reduction reactions play a significant role, the search for alternative materials that could replace expensive Pt-based cathodes has become an important challenge. In this regard, carbon materials (CMs) that are stable, electrochemically durable, and exhibit anti-corrosive performance show a significant potential to become widely used metal-free electrocatalysts^1^. In addition, such CMs may have a low price and sustainable character, especially when derived from natural, abundant, and renewable resources^2–4^.

Both economic and environmental considerations motivate the development of simple synthetic protocols for synthesizing CMs with inexpensive, widespread precursors that ideally provide simultaneous heteroatom doping and porosity. Various naturally occurring precursors, such as cellulose^5^, lignin^6^, wood^7^, coconut shells^8^, saccharides^9,10^, sugarcane bagasse^11^, spongin scaffolds^12^ and many others, can be transformed into CMs by biological, chemical, or thermochemical processes^3^. Also, low molecular weight compounds, such as amines, when converted into protic ionic liquids or salts (PILs/PSs), have received considerable attention as simple and versatile precursors that afford CMs with additional nitrogen doping^13^. These precursors can be synthesized at low cost in a straightforward reaction between an acid and an amine^14,15^ and then transformed via carbonization into products of a high value, such as CMs and nanomaterials. PILs, and PSs based on amines and abundant amino acids (available as waste proteins from agriculture and forestry) that showed large molecular diversity and high carbon content have been used as multifunctional carbon precursors.^16–18^ The protonation of amines and amino acids reduces their volatility and increases their thermal stability, leading to a reduced mass loss prior to the decomposition process, which facilitates carbon formation, resulting in increased carbon yield. The protonation of amines and amino acids with sulfuric acid^13,16,18^ and phosphoric acid^17^ yielded upon thermal treatment sustainable carbon residues with a moderate to high S_BET_ surface area and dual N,S/N,P-doping. For precursors that produced carbon residues with insufficient porosity, additional activation was applied to generate materials with good performance as electrodes in supercapacitors^16–18^.

High specific surface area (SSA) is an important feature of CMs, especially those utilized as catalysts, electrodes, or adsorbents. The common synthetic procedure to increase the SSA involves the use of a matrix, which is removed after the pyrolysis step^19–21^. The activation of nonporous CMs can also be achieved by treatment with KOH, NH_4_Cl, K_2_CO_3_, NaHCO_3_, or (NH_4_)_2_HPO_4,_ which are inexpensive and effective, yet other environmentally benign activators have also been explored, such as potassium oxalate^22^ and phytic acid (PA)^23^.

PA, widely distributed in nature in various seeds, grains, and nuts, is a sugar (inositol) derivative rich in phosphate groups. Upon thermal treatment (temperatures > 100 °C) in the presence of water, PA hydrolyzes into phosphoric acid (Eq. 1), which further transforms at 400–500 °C into P_2_O_5_ (Eq. 2), which is responsible for pore formation upon sublimation^24^. Pores are further formed during sublimation of red P that is formed from residual P_2_O_5_ (Eq. 3).

1 $${\text{C}}_{{6}} {\text{H}}_{{{18}}} {\text{O}}_{{{24}}} {\text{P}}_{{6}} + {\text{ 6H}}_{{2}} {\text{O }} = {\text{ 6H}}_{{3}} {\text{PO}}_{{4}} + {\text{ C}}_{{6}} {\text{H}}_{{{12}}} {\text{O}}_{{6}}$$

2 $${\text{2H}}_{{3}} {\text{PO}}_{{4}} = {\text{ P}}_{{2}} {\text{O}}_{{5}} + {\text{ 3H}}_{{2}} {\text{O}}$$

3 $${\text{P}}_{{2}} {\text{O}}_{{5}} + {\text{ 5C }} = {\text{ 2P }} + {\text{ 5CO}}$$

The carbonization of PA at 800 °C without any dopant or activator can yield material with an SSA as high as 1637 m^2^ g^−1^ ^25^. Additionally, PA serves simultaneously as a doping agent, providing CMs containing phosphorus atoms up to loadings of as much as 13.6% at 500 °C^25^, enhancing their electrical properties. P-doping can generate interaction with carbon atoms, resulting in redistribution of charge and spin density, favoring electrochemical reaction on the surface^1^. Taking advantage of the pore-generating and doping ability of PA combined with its sustainable character, PA-based carbon precursors have recently gained considerable attention^26–30^, which we summarized in a recent review^31^. Despite the natural origin of PA and its relatively simple production by extraction with aqueous acids, the strategies for the synthesis of phytic acid-derived P,N-doped carbons commonly involve using fossil-fuel-derived amines such as aniline^32^, *o*-phenylenediamine^33,34^, or 2,6-diaminopyridine^35^. Replacement of these petroleum-derived chemicals with sustainable alternatives can bring the transition to net-zero closer^36^.

We, therefore, capitalized on the well-established procedure for the simple carbonization of protic salts, replacing traditional mineral acids with PA. Moreover, we focused on the replacement of fossil-fuel-derived amines with amino acids to develop a series of fully bio-derived PA-based protic salts with a high molecular diversity (composed of various amino acids). For comparison, a series of amine-based protic salts was studied. We report the in-depth correlation between bio-derived protic salts and the properties of sustainable CMs afforded upon their one-step, facile carbonization. The advantages of using fully bio-derived protic salts over conventional H_2_SO_4_/H_3_PO_4_-based protic salts are elucidated and discussed.

Biomass-derived CMs commonly form brittle powders. These powders require blending with polymeric binders and loading onto conductive substrates, such as carbon cloth, for application as cathodes in fuel cells or metal-air batteries. The electrocatalyst is usually mechanically coated onto the substrate surface, which can often lead to detachment. To tackle the problem of the high brittleness of the protic salt-derived CMs, we hybridized as-prepared CMs with single-walled carbon nanotubes (SWCNTs) to afford flexible, electrically conducting, self-standing composites, due to the intertwined nature of the SWCNT. These materials were thoroughly evaluated as electrocatalysts in the oxygen reduction reaction. The substantial insight obtained during this study allows us to aim to develop a new generation of sustainable electrodes to improve a broad spectrum of essential chemical transformations.

## Results and discussion

### PA salts preparation and characterization

**Figure 1:**
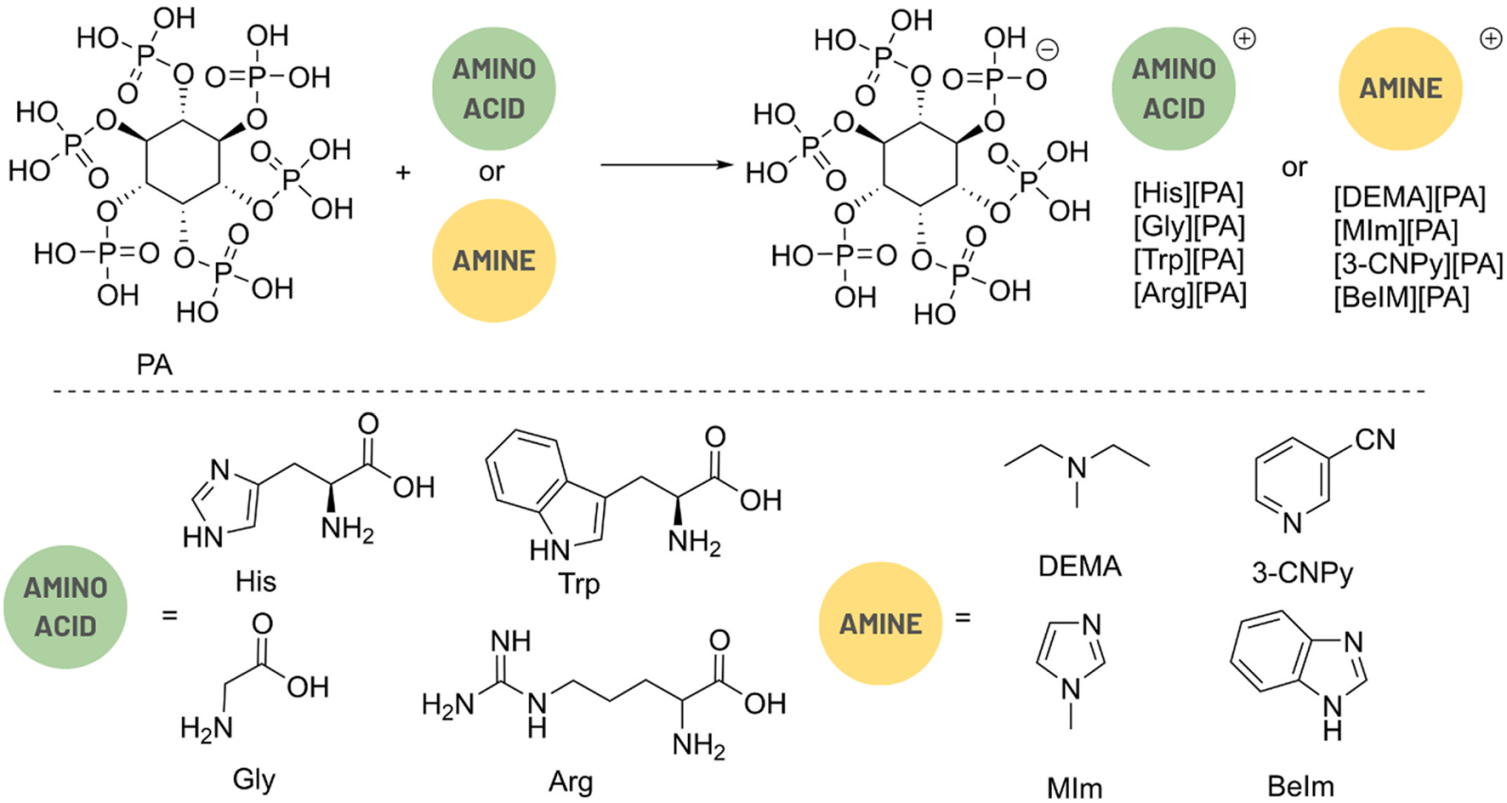
Synthetic route toward investigated protic salts of PA and amino acids/amines.

Bio-derived PA salts were prepared by a simple one-step reaction of PA with four amino acids (histidine (His), arginine (Arg), tryptophan (Trp), and glycine (Gly)) and four amines (1-methylimidazole (MIm), benzimidazole (BeIm), 3-pyridinecarbonitrile (3-CNPy), and N,N-diethylmethylamine (DEMA)) (Fig. 1). Salts comprising conventional mineral acid (sulfuric acid) and amino acids with heteroaromatic units, such as His or Trp, or N/S-terminal side chains, such as Arg, have been previously shown to provide CMs with a high carbon yield^16,37^. Additionally, salts of MIm, BeIm, 3-CNPy, DEMA and sulfuric acid were reported to give carbon materials with high nitrogen doping upon carbonization^13^.

The salts were prepared using various molar ratios of PA to amino acid/amine, i.e., 1:1, 1:2, 1:3, 1:4, and 1:6. The differing compositions of the salts were characterized by ^1^H NMR spectroscopy (S1 and S2). The signals corresponding to aromatic protons in the imidazole moiety of histidine (δ = 7.7 ppm; δ = 7.0 ppm) were strongly deshielded for [His][PA] 1:1 (δ = 8.6 ppm; δ = 7.3 ppm), similarly to the signals corresponding to the aliphatic protons (δ = 3.9–4.0 ppm; δ = 3.2–3.1 ppm for histidine; δ = 4.3 ppm; δ = 3.3 ppm for [His][PA] 1:1), suggesting salt formation. Furthermore, on increasing the proportion of His in the salt composition, further deshielding of the signals was observed for [His][PA] 2:1 compared to [His][Pa] 1:1. However, for salts with a much higher content of His ([His][PA] 4:1, [His][PA] 6:1), the shielding of signals, especially those corresponding to aliphatic protons, was observed, most probably caused by the presence of unprotonated His in the mixture. Salts based on MIm followed this trend (S2).

**Figure 2:**
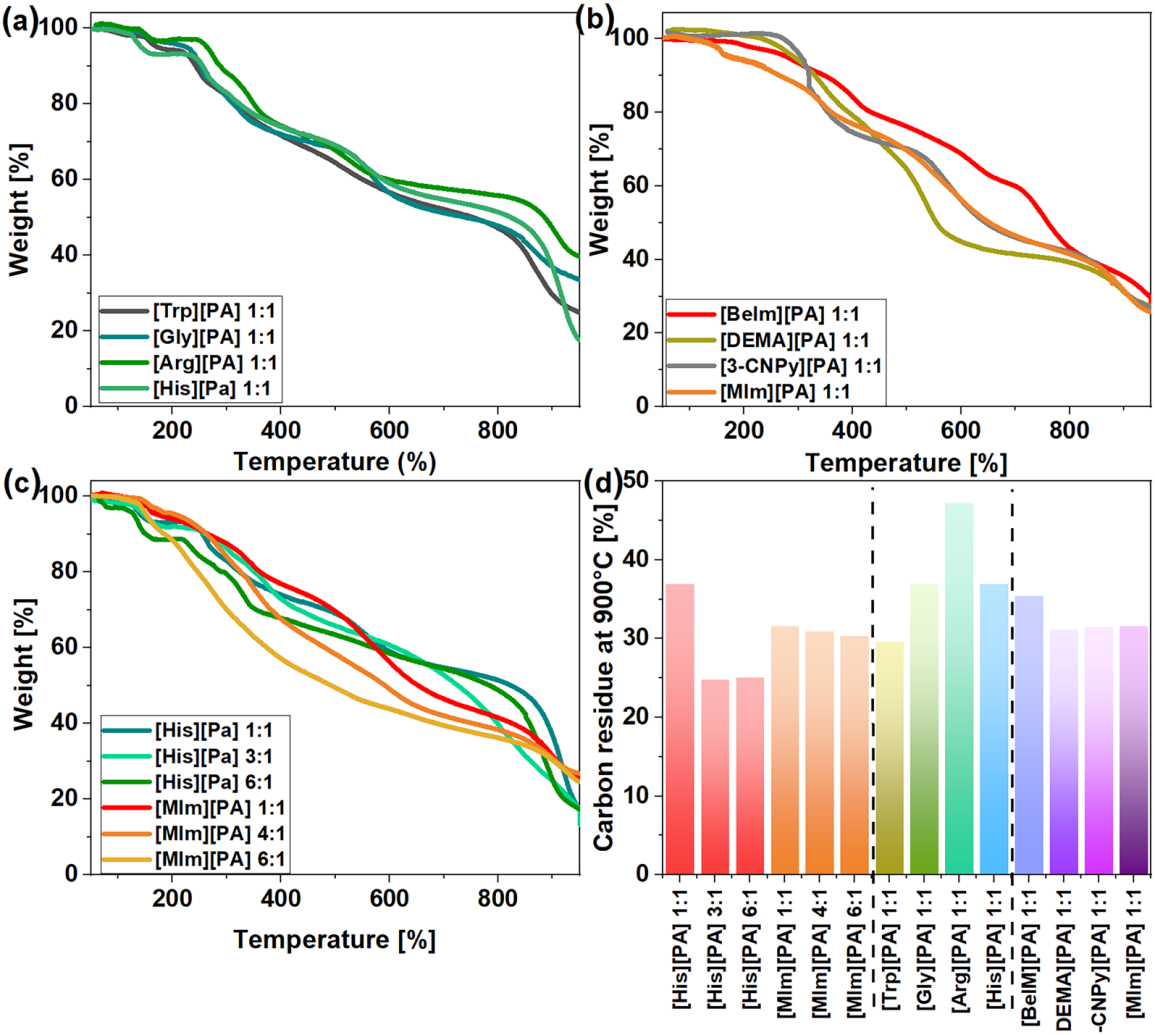
Thermogravimetric analysis curves of PA-derived salts (**a**) based on amino acids, (**b**) based on amines, (**c**) with various molar compositions. (**d**) Comparison of carbon residues from PA-derived salts at 900 °C.

The salt composition influenced its thermal stability and char-forming ability, as shown by [His][PA] and [MIm][PA] (Fig. 2c). The highest carbon residues were yielded by salts with a molar composition of PA to amino acid/amine of 1:1. For [His][PA]1:1, the residues reached 37% at 900 °C (51.4% at 800 °C), while for [MIm][PA]1:1 they amounted to 33.1% (41.5% at 800 °C). The final residues were higher than that produced by neat His, 36% (40.4% at 800 °C) (S3), while MIm decomposed completely at 800 °C^38^. The achieved yields were also considerably higher than that achieved with neat PA (30.0% at 800°C^39^), thus demonstrating the excellent char-forming ability of these simple bio-derived PA salts. The thermogravimetric analyses of the protic salts with the remaining amino acids and amines are shown in Figs. 2a, b, respectively. Amino acids converted into PA salts gave carbon chars in higher yields (> 30% at 900 °C, for the Arg salt it reached as high as 47.2% (55.9% at 800 °C) than the corresponding pristine amino acids (Trp, Gly, and Arg) for which yields of up to 20% were reached^37^. Even though neat Arg showed a worse carbon-forming ability than His^40^, its protic salt with PA ([Arg][PA]1:1) left more char residues (47.2%) than its His-derived analog (36.9%) (Fig. 2d). Carbon residues produced from the corresponding amine-derived protic salts were lower, reaching up to 35.4% at 900 °C for the BeIm-derived salt ([BeIm][PA]). The exception from this trend showed only [Trp][PA] which gave the least carbon chars from the amino acid-based salts (29.6%).

### PA salts carbonization

The amino acid-derived salts and amine-derived salts were carbonized at given temperatures (800, 900, and 1000 °C) to give CMs with specific properties. The type of amino acid/amine as well as the salt composition highly affected the morphology, porosity and composition of the obtained CMs (Table 1).

**Table 1:** Structural properties and composition of the protic salt-derived CMs.

Carbon precursor	Carbonization temperature (°C)	S_BET_ (m^2^ g^−1^)	S_mic_ (m^2^g^−1^)	V_tot_ (cm^3^ g^−1^)	V_mic_ (cm^3^ g^−1^)	Pore size (nm)	Elemental analysis data			XPS analysis data
							N (%)	C (%)	H (%)	P (%)
[His][PA] 1:1	800	537	253	0.27	0.11	2.6	5.2	59.6	2.6	3.08
[His][PA] 3:1	800	738	346	0.36	0.15	2.6	4.8	59.1	2.7	3.66
[His][PA] 6:1	800	2	2	0.01	< 0.01	20.8	6.2	53.5	2.2	5.63
[MIm][PA] 1:1	900	538	373	0.30	0.17	5.4	6.3	61.5	1.7	5.59
[MIm][PA] 4:1	900	736	406	0.40	0.17	3.8	4.2	67.3	1.3	6.83
[MIm][PA] 6:1	900	346	190	0.08	0.19	3.7	2.6	40.9	0.9	4.41
[Trp][PA] 1:1	800	118	3	0.13	< 0.01	4.2	2.8	48.4	2.4	7.32
[Arg][PA] 1:1	800	792	465	0.51	0.21	7.1	2.8	59.4	1.9	3.32
[Gly][PA] 1:1	800	149	25	0.30	0.01	12.3	2.6	50.8	2.1	6.61
[BeIm][PA] 1:1	900	697	174	0.07	0.28	3.97	2.2	58.4	1.6	4.07
[3-CNPy][PA] 1:1	900	1177	56	0.67	0.02	2.6	2.2	63.8	1.4	4.93
[DEMA][PA] 1:1	900	410	343	0.22	0.16	6.4	1.9	63.8	1.4	3.96

Salts derived from Arg and 3-CNPy gave rise to CMs with high specific surface areas of 792 and 1177 m^2^ g^−1^, respectively, indicating that highly porous CMs can be synthesized using simple bio-derived PA salts in a one-step carbonization, without using any additional template or post activation method. The appreciable specific surface area of the corresponding CMs can be ascribed to the superior pore-generating ability of the PA (the carbonized corresponding salt of 3-CNPy and sulfuric acid ([3-CNPy][HSO_4_]) yielded material with an S_BET_ of scarcely 6 m^2^ g^−1^ at 1000 °C)^13^.

**Figure 3:**
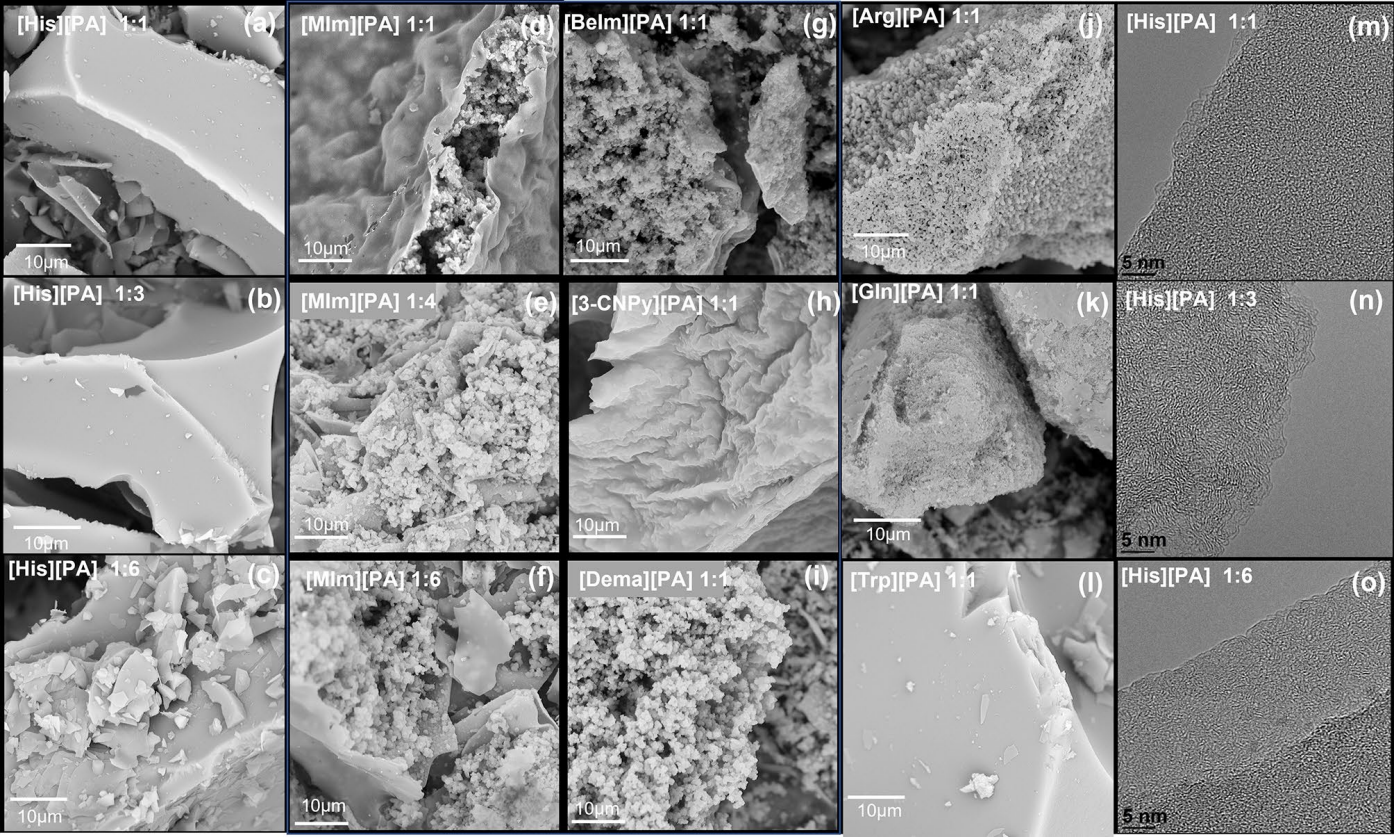
SEM images of CMs obtained from amine/amino acid-derived protic salts: (**a**) [His][PA] 1:1, (**b**) [His][PA] 1:3, (**c**) [His][PA] 1:6, (**d**) [MIm][PA] 1:1, (**e**) [MIm][PA] 1:4, (**f**) [MIm][PA] 1:6, (**g**) [BeIm][PA] 1:1, (**h**) [3-CNPy][PA] 1:1, (**i**) [DEMA][PA] 1:1, (**j**) [Arg][PA] 1:1, (**k**) [Gly][PA] 1:1, (**l**) [Trp][PA] 1:1. HRTEM images of CMs obtained from amino acid-derived protic salts: (**m**) [His][PA] 1:1, (**n**) [His][PA] 1:3, (**o**) [His][PA] 1:6.

The investigated bio-derived protic salts gave various microstructures upon carbonization, indicating the predominant influence of the amine/amino acid type on the structure and porosity, therefore influencing the possible future applications of the CM. The representative SEM images of the CMs derived from the various amino acids and amines are depicted in Fig. 3. Amino acids such as Arg and Gly, and amines such as BeIm, DEMA, and MIm provided CMs with a porous, spherically shaped agglomerated particle-like structure with the size of individual particles varying from approximately 10 to 100 nm (Fig. 3d, g, f, j, k). For the MIm-derived CM, the spherical particles were sandwiched by two highly wrinkled graphene-like sheets (Fig. 3d). Similar sandwiched structures were reported for materials obtained by direct microwave treatment of PA^41^. The wrinkled structure was previously ascribed to the result of P doping and local geometrical distortion in the carbon network induced by the larger diameter of P atoms compared to C atoms^41^. According to Barrett–Joyner–Halenda (BJH) pore size and volume analyses, despite a similar morphology, the obtained materials differed in terms of S_BET_, ranging from 149 m^2^/g for Gly to 792 m^2^/g measured for its Arg-analog. Such variation, to some extent, can be explained by the variable proportion of the smallest pores, the presence of which cannot be assessed from SEM images.

By contrast, the 3-CNPy-, Trp-, and His-derived materials showed distinctly different microstructures by SEM analysis—compact and surrounded by flat surface, without distinct spherical nanoparticles, and highly wrinkled in the case of the 3-CNPy-derived CM (Fig. 3a, b, h, l) (the presented SEM images are representative, indicating that no spherical nanoparticles were observed for any of these analyzed samples). This highly wrinkled morphology contributed to the highest specific surface area of all the investigated materials (S_BET_ of 1177 m^2^/g), while the Trp-derived CMs exhibited an S_BET_ of 118 m^2^/g. In the case of His-derived CMs, a surprising variation in the S_BET_ values was observed depending on the salt composition. Increasing the molar ratio of His to PA from 1:1 to 1:3, the S_BET_ increased from 537 to 738 m^2^/g. However, a further increase in the amino acid/amine content in the salt composition resulted in a large drop in the specific surface area to 2 m^2^/g (the same trend, yet less pronounced, was observed for the MIm-derived series). Since the microstructures observed using SEM looked similar (Fig. 2a–c) and did not explain these distinct changes, more detailed TEM analyses were performed. The obtained high-resolution HRTEM images showed no noticeable differences at the nanoscale (Fig. 3 m–o). Therefore, the significant difference in S_BET_ values (537, 738, and 2 m^2^/g), which could not be justified by a difference in morphology, must have been caused by structural changes at the micropore level (< 2 nm), which, due to the resolution, were not visible even in the HRTEM images. The large specific surface area resulted from the presence of micropores. The increase in the content of amino acids/amine in the salt composition meant that these smallest pores were no longer available. They may either have been blocked (this was not confirmed by the images obtained), or there were structural changes leading to slight changes (reduction) in the distance between the carbon layers. Although these changes were too small to be observed in the TEM images, they still effectively prevented nitrogen sorption.

**Figure 4:**
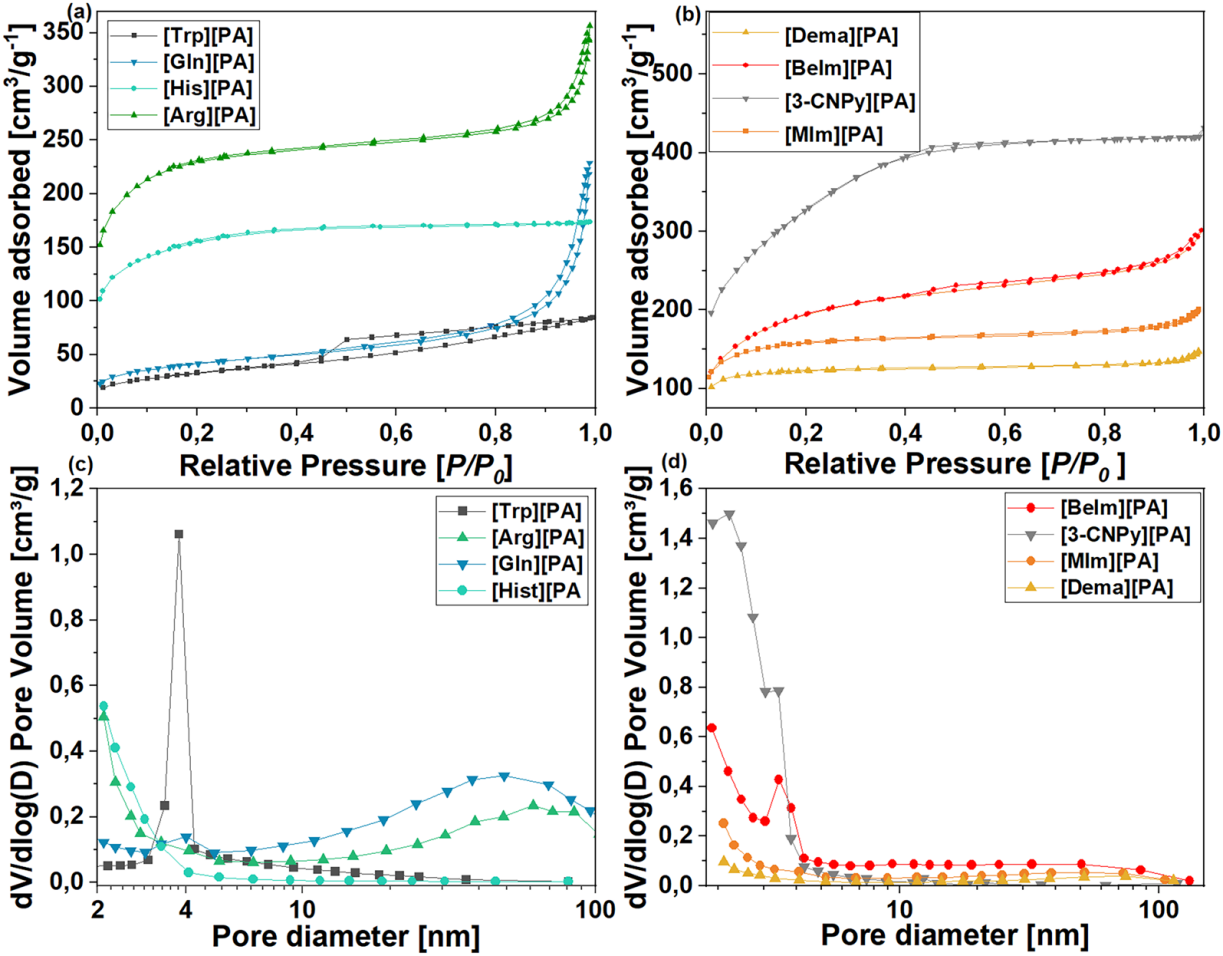
N_2_ gas adsorption/desorption isotherms and pore-size distribution of carbons derived from (**a**,**c**) salts of PA and amino acids; (**b,d**) salts of PA and amines.

The textural properties of the bio-derived CMs were further assessed by N_2_ sorption isotherms, shown in Fig. 4. The porosity of the materials mainly originated from micropores and small mesopores < 10 nm, which is common for the PA-derived CMs^25,26,28^. The materials with compact surfaces observed with SEM microscopy (derived from [3-CNPy][PA] and [His][PA]) exhibited smaller mesopores (< 4 nm) as confirmed with the pore-size distribution analysis (Fig. 4c, d) but mostly larger than 2 nm, especially for CM derived from [3-CNPy][PA] 1:1 what is expressed in its S_mic_ of only 56 m^2^ g^−1^. The material obtained from [Trp][PA], even though revealed similar morphology under the SEM analysis, it also showed larger pores centered at 4 nm. In contrast to the CMs derived from 3-CNPy, Trp, and His, the materials generated from other amino acids/amines revealed the presence of larger mesopores (noted by SEM and pore-size distribution analyses). Besides differences in the size of pores, all the the materials demonstrated type IV isotherm curves with narrow H4 hysteresis loops, indicative of mesoporosity. The investigated bio-derived precursors afforded CMs with triple N,P,O doping. The phytate moiety contributed to a relatively high content of oxygen and phosphorus in the as-prepared CMs due to the decomposition of multiple oxygen- and phosphorus-rich functional groups, while the amine/amino acid moiety acted as a nitrogen precursor. The composition of the as-prepared CMs regarding the carbon and nitrogen content was determined using elemental analysis, while the phosphorus content was determined using XPS analysis. The type of amino acid/amine influenced the nitrogen content in the final materials, which ranged from 1.9 to 6.3 wt%. Interestingly, most of the amino acid-derived salts yielded materials with an N content in the range of 2.6 to 2.8%. The exception was the His-derived salt, which afforded a CM with a N content of 5.2% (measured with the [His][PA] salt with a 1:1 acid:amino acid ratio). Similarly, most of the amine-derived analogs produced CM with a nitrogen content in the range of 1.9 to 2.2%. In this series, the exception was the MIm-derived salt, which afforded a CM with 6.3% nitrogen, which was the highest doping among all the investigated CMs. Increasing the amine/amino acid content in the salt composition did not translate to higher nitrogen doping. This counterintuitive result can be explained by the relatively greater decomposition of the precursors possessing a higher content of the amine/amino acid, most probably caused by the presence of unprotonated amine/amino acid in their composition, which are known to decompose more easily than their salt analogs. On the other hand, an increased amine/amino acid content in the salt composition translated into an increased phosphorus content in the CMs. For most of the CMs derived from the salts with a 1:1 molar ratio, the P-doping did not exceed ∼5% (the exception were CMs derived from Gly and Trp derivatives for which the P-doping reached up to 7%). The discrepancies in P-doping among specific samples may result both from the sample’s specific properties and the analytic method used for the P content determination—the XPS method only provides information related to the near-surface region of the CMs, not the overall heteroatom content, as was measured for N and O using EA; therefore, the local aggregation of heteroatoms cannot be excluded.

**Figure 5:**
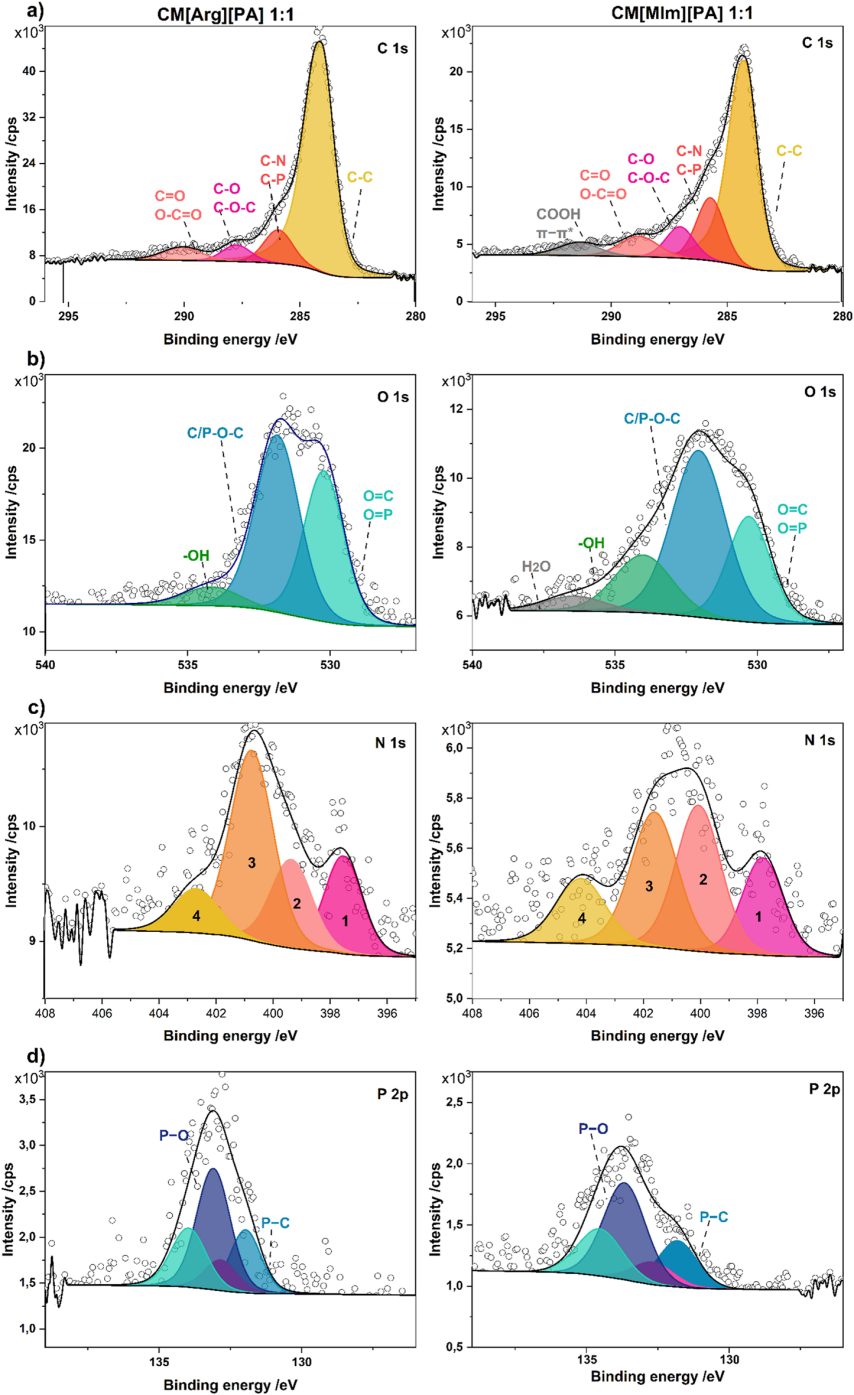
(**a**) C 1s, (**b**) O 1s, (**c**) N 1s, and (**d**) P 2p XPS spectra for CM[Arg][PA] 1:1_800 and CM[MIm][PA] 1:1_900.

The chemical environment of the elements in the near-surface region of the CMs was further determined by XPS. The representative spectra measured for CMs prepared from [Arg][PA] 1:1 and [MIm][PA] 1:1 are presented in Fig. 5. The primary component of all species was carbon, the high-resolution C 1s spectrum of which was decomposed into four or five main peaks for CMs derived from [Arg][PA] 1:1 and [MIm][PA] 1:1, respectively. The major contribution was assigned to the presence of C–C (at ∼284.2 eV, which points to the dominating sp^2^ hybridization^42,43^). This component was fitted with the asymmetric product of Gaussian and Lorentzian functions with an exponential tail to represent the contribution from the C–C satellite. Components were then assigned to C–N/C–P (∼285.9 eV), C–O/C–O–C (∼287.4 eV), and C=O/O−C=O (∼290.1 eV). The additional COOH and weak π−π* satellite were noted on the high-binding-energy side of the C 1s spectrum (291.4 eV) for [MIm][PA] 1:1. Among the heteroatoms present in the CMs (N,P, and O), the content of oxygen was the highest. The O 1s spectra were decomposed (basing on the constituents’ electronegativity values and additionally verified with NIST database^44^) into three main peaks, indicating the presence of O=C/O=P (∼530.2 eV), C/P–O–C (∼532.0 eV), and –OH (∼534.3 eV) groups in both materials. The additional fourth peak found for [MIm][PA] 1:1 at 536.5 eV was ascribed to physisorbed H_2_O. The N 1s spectrum was decomposed into four peaks corresponding to pyridinic N (∼397.8 eV) (1), pyrrolic N (∼399.3 eV) (2), graphitic-type quaternary N (∼400.7 eV) (3), and oxidized N species (∼402.7 eV) (4). Finally, the P 2p spectrum showed two peaks, corresponding to P–C (131.9 eV) and P–O (133.1 eV), both with their respective spin–orbit (splitting value of 0.87 eV ^45^) counterparts.

### PA salts vs. sulfuric acid salts as precursors for CMs

**Figure 6:**
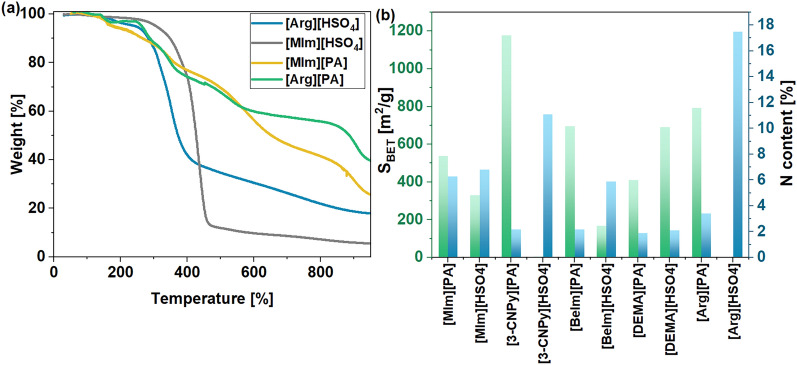
(**a**) Thermogravimetric curves of sulfuric acid-derived protic salts and their corresponding PA analogs with 1:1 molar ratio; (**b**) comparison of specific surface area and nitrogen content of CMs synthesized at 900 °C from sulfuric acid and PA derivatives with a 1:1 amine/amino acid: acid molar ratio. The values of S_BET_ and N content for CMs synthesized from sulfuric acid salts are from reference ^13^.

The materials derived from the salts of PA had, in most cases, an increased surface area compared to their sulfuric acid analogs (Fig. 6b) (the CM derived from [3-CNPy][PA] exhibited an S_BET_ of as much as 1177 m^2^/g, while its mineral acid analog [3-CNPy][HSO_4_] gave an S_BET_ of only 6 m^2^/g). Moreover, PA-derived salts showed significantly higher char-forming ability (for Arg- and MIm-derived salts at least two times higher, as depicted in Fig. 6a) and additional P-doping, ranging from 3.08 to 7.32%, depending on the amino acid/amine precursor used.

Regarding nitrogen content, the doping level depended to a great extent on the precursor, i.e. amino acid/amine. Similar results were achieved using mineral and bio-based analogs for their MIm derivatives (nitrogen content of 6.8% and 6.3% was reported for [MIm][HSO_4_] and [MIm][PA] derived CMs, respectively) or DEMA derivatives (a nitrogen content of 6.8% and 6.3% was reported for [DEMA][HSO_4_] and [DEMA][PA] derived CMs, respectively), but significantly decreased nitrogen doping for CMs derived from PA salts such as [3-CNPy][PA] and [BeIm][PA] was noted (Fig. 6b). Nevertheless, the bio-derived origin of PA and its simple production method (by biomass extraction)^46^ pave the way toward the development of inexpensive, sustainable, and functional materials. Moreover, it is possible to control the properties of the derived CMs by using a carbon precursor mixture of different PA salts (preferably providing both a high surface area and high nitrogen/phosphorus doping) in various molar ratios^10^.

### Electrochemical activity in ORR

**Figure 7:**
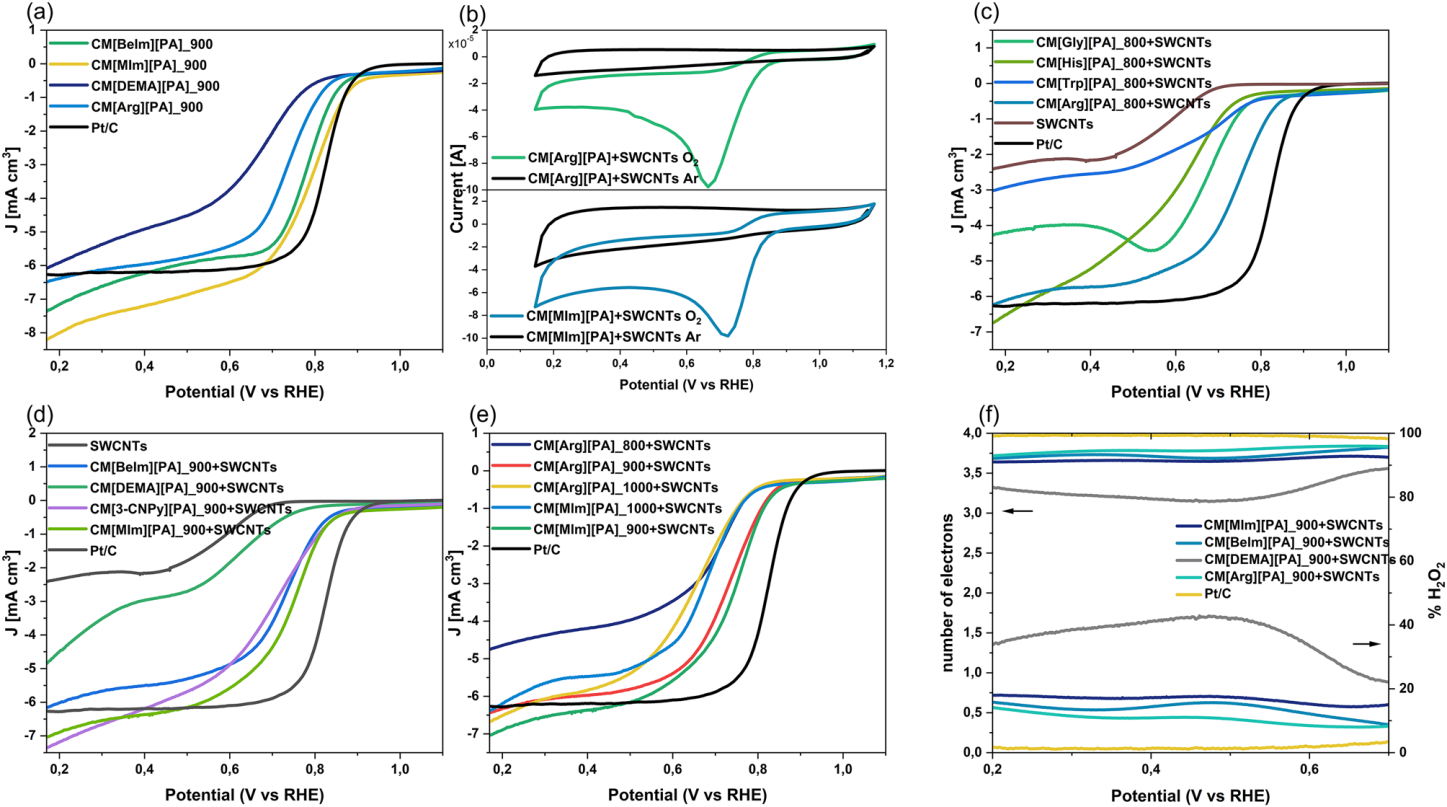
(**a**) LSV recorded for CMs derived from protic salts, (**b**) CV curves recorded for composites CM[Arg][PA]_900 + SWCNT (50/50%wt) and CM[MIm][PA]_900 + SWCNT (50/50%wt), (**c**) LSV recorded for composites of CMs derived from amino acid based protic salts and SWCNTs (50/50%wt), (**d**) LSV recorded for composites of CMs derived from amine based protic salts and SWCNTs (50/50%wt), (**e**) LSV recorded for composites of CMs carbonized at various temperatures and SWCNTs (50/50%wt) deposited on a GC electrode in O_2_-saturated 0.1 M KOH at a scan rate of 50 mV/s. (**f**) Electron-transfer numbers and peroxide yields (H_2_O_2_%) calculated for composites of CMs derived from amino acid/amine based protic salts and SWCNTs (50/50%wt) from RRDE measurement registered in O_2_-saturated 0.1 M KOH at a scan rate of 0.01 V/s.

The CMs prepared by one-step carbonization gave noticeable electroactivity toward the oxygen reduction reaction (ORR), which was strictly related to the precursors' structure (Fig. 7a). The onset potentials for the ORR process measured by linear sweep voltammetry (LSV) polarization curves recorded at a rotating speed of 1600 rpm were approximately 0.88, 0.86, 0.82, and 0.78 V (vs. RHE), for [MIm][PA]_900, [BeIm][PA]_900, [Arg][PA]_900, and [DEMA][PA]_900 respectively, indicating that the highest electrocatalytic activity was exhibited by CMs with high surface area and aggregated particle-like structure. Nevertheless, the surface area and morphology are not the only factors influencing the electrocatalytic activity, as observed for [MIm][PA]_900. The other factor is heteroatom doping (almost 3 times higher for [MIm][PA]_900 than for [BeIm][PA]_900). The film-forming properties of bio-CMs with no distinct pores noted by SEM analysis (derived from [Trp][PA], [His][PA], and [3-CNPy][PA]) were insufficient to prepare inks for electrochemical characterization. All the pristine CMs suffered from high brittleness no matter which PA salt was used to synthesize them, which is a common weakness of biomass-derived CMs.

**Figure 8:**
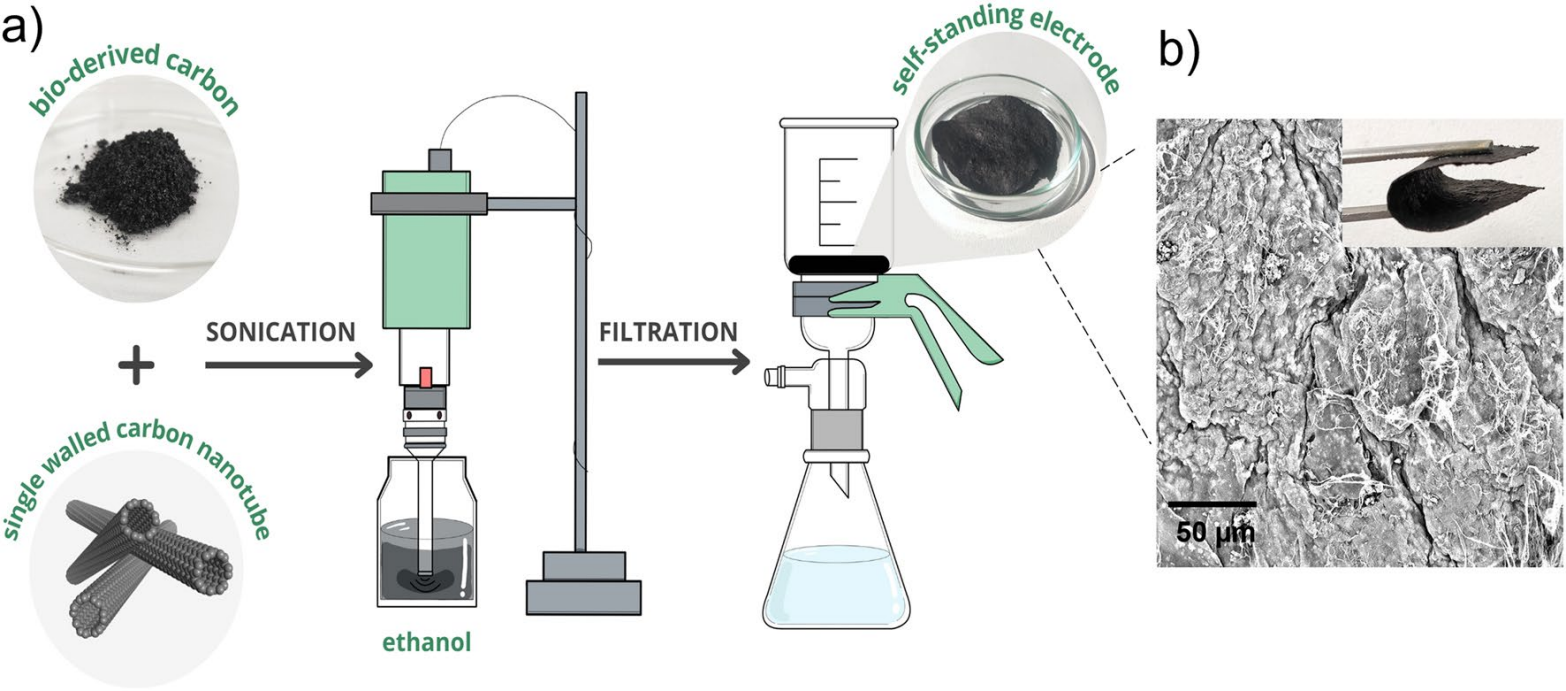
(**a**) Schematic illustration of the preparation process for the flexible bio-derived composites. (**b**) SEM image of CM[MIm][PA]_SWCNTs 50/50%wt.

To improve the textural properties of the bio-derived CMs, they were combined with SWCNTs by a simple procedure depicted in Fig. 8 to afford thin films. The composites were prepared using 33, 50, and 75% wt of SWCNTs with respect to CMs. Using 33 wt% of the SWCNTs was insufficient to form a flexible, free-standing composite, in contrast to films prepared with 50 or 75 wt%, for which SEM images are shown in Fig. S4. The intertwined nature of the SWCNT ensembles held by numerous van der Waals forces provided a flexible scaffold to which the brittle PA-derived CMs could attach, so robust thin free-standing films of ca. 100 μm in thickness were formed from such composites^47^.

The 50% compositions of bio-derived CMs with SWCNTs were prepared in the form of inks that were applied to a disk electrode. The electrocatalytic activity of the prepared composites was confirmed by cyclic voltammetry (CV) using a three-electrode system in 0.1 M KOH solution (Fig. 7b). In the O_2_-saturated solutions, strong cathodic peaks appeared at 0.72 V and 0.70 V for representative CM[MIm][PA] and CM[Arg][PA], respectively, associated with the irreversible reduction of oxygen, in contrast to Ar-saturated solutions. Unlike pristine bio-derived carbons, bio-CMs combined with SWCNTs all afforded inks for the RRDE measurements (Fig. 7c–e). The material composed of 50% wt SWCNTs showed an onset potential at 0.83 V, which is approximately 0.05 V more negative than that of pristine CM[MIm][PA]. The pristine SWCNTs had low electrochemical activity toward ORR with the onset potential for the ORR process at 0.69 V (vs. RHE) (Fig. 7c, d); therefore, the selection of a suitable SWCNT loading to prepare a composite was a compromise between flexibility and electrochemical activity (the inclusion of SWCNTs was also essential due to their exceptional electrical conductivity^48^, which enables facile charge propagation throughout the whole composite). The catalytic activities of the composites containing CMs synthesized from various amino acid or amine protic salts are depicted in Fig. 7c, d, respectively. The onset potential for the ORR was the most positive (approximately 0.82–0.84 V) for composites containing CM[3-CNPy][PA], CM[MIm][PA], CM[Arg][PA], and CM[BeIm][PA], indicating the positive influence of the high surface area of the pristine CMs. The level of nitrogen in the precursors also contributed to the higher catalytic activity of the composites, as shown for CM[MIm][PA], which, despite having a lower surface area (538 m^2^ g^−1^) than CM[BeIm][PA] (697 m^2^ g^−1^), exhibited a higher nitrogen content (6.3% vs. 2.2%), which contributed to its enhanced catalytic performance.

The next parameter that can significantly affect the electrocatalytic performance of doped CMs is the carbonization temperature^49^. As depicted in Fig. 7e, protic salts carbonized at 900˚C yielded materials with the best electrocatalytic performance for both amine- and amino acid-derived CMs in terms of their particularly positive onset potentials and their large diffusion-limiting current density.

To further investigate the electron transfer number (n) and the peroxide yield (H_2_O_2_%) of the composites, RRDE measurements were performed in 0.1 M KOH solutions saturated with O_2_ (Fig. 7f). In the potential range of 0.2–0.7 V (vs. RHE), the average electron transfer numbers were calculated to be 3.65 for CM[MIm][PA]_SWCNTs and 3.7–3.8 for CM[Arg][PA]_SWCNTs and CM[BeIm][PA]_SWCNTs, indicating that the ORR proceeded mainly through a four-electron process (O_2_ to H_2_O). In turn, the peroxide yields measured for CM[Arg][PA]_SWCNTs and CM[MIm][PA]_SWCNTs were less than 14% and 17%, respectively. For the composites made of bio-CMs with significantly lower S_BET,_ such as CM[DEMA][PA], the average electron transfer numbers were lower (approaching 3.1), indicating the enhanced occurrence of the two-electron process (O_2_ to H_2_O_2_). Finally, we measured the conductivity of the films composed of PA-derived CMs and SWCNTs. The film composed of 50% wt SWCNTs and 50% wt PA-derived CMs showed high conductivity of 1234 S/cm. In comparison, a film composed exclusively of SWCNTs exhibited a conductivity of 889 S/cm, highlighting the beneficial impact of incorporating doped carbons into the composite.

The above results confirmed the significant activity of the composites toward ORR. Moreover, their free-standing structure and excellent conductivity make them suitable for direct application as a working electrode (WE) without using any binder (e.g. the typically employed Nafion) or substrate, which can negatively affect the performance.

## Conclusion

Protic salts, recognized as low-cost precursors for CMs, were prepared using PA as a replacement for the traditional sulfuric acid and used as precursors for sustainable N,P,O-doped CMs. The PA played the role of a pore-generating agent, providing materials with enhanced surface area (up to 1177 m^2^ g^−1^) without the need to use a hard template in the carbonization process or any additional post-activation method. Moreover, the PA salts had a higher char-forming ability than their mineral acid analogs and provided carbon materials with simultaneous phosphorus doping. By precisely choosing the amine/amino acid for the synthesis of protic salts, it was possible to tune the properties of bio-derived CMs in terms of N content, porosity, and carbon yield. Moreover, through hybridization of bio-derived carbons with SWCNTs, highly flexible composites could be prepared, ready for application as a working electrode for electrocatalytic ORR in the absence of any substrate or binder. The self-standing composites produced, despite having lower electrocatalytic activity than commercial Pt/C catalysts, challenge them in terms of sustainability, flexibility, and cost.

## Methods and materials

### Materials

PA (50% aqueous solution), His, Arg, Trp, Gly, MIm, BeIm, 3-CNPy, DEMA, and ethanol were purchased from Sigma-Aldrich and used as received. SWCNTs (Tuball™) were purchased from OCSiAl. Properties of SWCNTs: diameter distribution: 1.6 ± 0.4 nm, length: > 5 μm, aspect ratio: ca. 3000, specific surface area: > 500 m^2^/g.

### Synthesis of protic salts

In a typical procedure, to a 50% aqueous solution of PA (1 mol eq.), placed in a round-bottomed flask in an ice bath, an appropriate amount of base/amino acid (1–6 mol eq.) was added dropwise (the amino acid was previously dispersed in water). After the completion of the addition, the mixture was vigorously stirred for 1 h at 55 °C, followed by water removal using a rotary evaporator and drying under high vacuum for 6 h (60 °C, 2–10 bar). As an example, the salt derived from PA and His mixed in the molar ratio of 1:1 is abbreviated as [His][PA] 1:1.

### Synthesis of CMs

The amino acid-derived salts and amine-derived salts were heated from 25 °C to 800, 900 or 1000 °C at a rate of 10° C/min with a holding time of 2 h in a tube furnace under an Ar atmosphere to give CMs. As an example, the CM derived from the salt [His][PA] 1:1 at 800 ˚C is abbreviated as CM[His][PA] 1:1_800.

### Free-standing film preparation procedure

The free-standing films were prepared by modifying a procedure reported by us previously^50^. Briefly, protic salt-derived CMs (50 mg) were mixed with SWCNT powder (25, 50, or 75% wt) in ethanol (50 mL), followed by sonication at 100% amplitude for 180 s (Hielscher UP200St, Germany). The obtained dispersion was filtered off using a vacuum through a PTFE membrane. The low adhesion of the produced films to this substrate allowed feasible delamination of the films to make them self-supporting. The preparation process is illustrated in further parts of the manuscript. As an example, the film derived from CM[His][PA] 1:1_800 is abbreviated as CM[His][PA] 1:1_800 + SWCNTs.

### Characterization

The ^1^H NMR spectra were recorded in D_2_O at 400 MHz (Agilent spectrometer). Thermogravimetric analysis (TGA) was performed using a Linseis STA PT1600 thermobalance (Selb). Samples (approximately 10–20 mg) were heated from 25 to 900 °C at a rate of 10 °C /min in alumina crucibles under a dynamic nitrogen flow of 80 mL/min. The morphologies of the carbon materials were characterized with a scanning electron microscopy (Phenom Pro Desktop SEM). TEM investigations were carried out with an FEI Titan 80–300 S/TEM microscope. The preparation of samples was performed as follows: the samples were crushed in an agate mortar, the obtained powders were dispersed in ethanol using an ultrasonic bath, and then droplets of these prepared dispersions were placed onto a carbon-coated lacy substrate supported by a copper grid and dried at room temperature. HRTEM imaging was carried out at 300 kV, and the images were recorded with a 1 s exposure time to avoid radiation damage of the samples.

Nitrogen sorption isotherms were obtained using a Micromeritics ASAP 2420 M instrument at − 196° C. Prior to the experiments, the samples were out-gassed at 200 °C and 1.33 × 10^–3^ Pa for 5 h. The specific surface areas were calculated using the Brunauer–Emmett–Teller (BET) method. The pore size distribution was obtained using the Barrett–Joyner–Halenda (BJH) method with the Harkins and Jura/ Faas correction. Elemental analyses (EA) were carried out using a Perkin Elmer 240 elemental analyzer.

X-ray photoelectron spectroscopy (XPS) measurements were carried out with an ultra-high-vacuum multi-chamber experimental setup comprising a PREVAC EA15 hemispherical electron energy analyzer fitted with a 2D multi-channel plate detector. The system base pressure was 9 × 10^–9^ Pa, and an Mg-Kα X-ray source (PREVAC dual-anode XR-40B source, excitation energy = 1253.60 eV) was used to excite the sample. The pass energy was set to 200 eV for the survey spectra collection, with a scanning step of 0.9 eV, or 100 eV for high-resolution energy regions, with a scanning step of 0.05 eV. All measurements were performed with a normal take-off angle and a curved analyzer exit slit (0.8 × 25 mm) selected for the highest energy resolution. The binding energy scale of the analyzer was calibrated to the Au 4f_7/2_ (84.0 eV) region of a gold-covered sample placed on the same sample stage^51^. The acquired spectra were fitted using CasaXPS^®^ software (version 2.3.25). The components were fitted with a sum of Gaussian (30%) and Lorentzian (70%) functions, while a Shirley function was applied for the background subtraction.

Electrochemical measurements were carried out using Autolab PGSTAT 204. A conventional three-electrode system was employed with a glassy carbon (GC) disc electrode (EDAQ, 1 mm diameter) or a GC-disc with a platinum ring (Autolab, 5 mm diameter) serving as a working electrode for RRDE measurements, a GC rod as a counter electrode, and an Ag/AgCl reference electrode. The substrate surface was polished with alumina and rinsed with deionized water prior to use. For determination of the catalytic performance of CMs derived from protic salts, the specific CM (5 mg) was dispersed by ultrasonication in a solution containing 25 μL of aqueous Nafion and 250 μL of ethanol. For determination of the catalytic performance of the composite materials, the inks were prepared as follows: 5 mg of protic salt-derived CM were mixed with 5 mg of SWCNTs in 50 μL of aqueous Nafion and 25 mL of ethanol, followed by sonication for 3 min at 100% amplitude (Hielscher UP200St, Germany). The solvent was evaporated to a constant volume of 4 mL by stirring the dispersion at 200 rpm at 110 °C. The resulting ink was dropped onto the GC surface (20 μL) and left for solvent evaporation, which yielded catalyst load of 0.254 mg/cm^2^ for LSV experiments. The measurements were conducted in 0.1 M KOH electrolyte solution. For the RRDE experiments (Autolab RRDE Instrument), the ring potential was held constant at 0.5 V vs. the reference electrode. The results of the RRDE experiments were further used for electron transfer numbers (n) and peroxide yield [Y (%)] determination using Eqs. (4) and (5), respectively:

4 $$n=4\times \frac{{I}_{d}}{\frac{{I}_{r}}{N}+{I}_{d}},$$

5 $$Y=200\times \frac{{I}_{r}/N}{\frac{{I}_{r}}{N}+{I}_{d}}$$

in which I_r_ and I_d_ are the disk and ring currents, respectively, and N is the ring collection efficiency (24.9%).

Electrical conductivity of the material was measured using a four-probe technique with a source meter (Keithley 2450 SourceMeter). The obtained conductance values were recalculated to conductivity by considering the dimensions of the sample. The thickness was determined using a micrometer.

### Supplementary Information

Supplementary Figures.

## Data Availability

The datasets used and/or analysed during the current study available from the corresponding author on reasonable request.
