# Supplementary material for: Bio-based protic salts as precursors for sustainable free-standing film electrodes
Source: Sci Rep. 2024 May 15;14:11106. doi: 10.1038/s41598-024-61553-x (PMC11096361; doi:10.1038/s41598-024-61553-x)
Supplement: Supplementary file 1 — Supplementary Figures. [file 41598_2024_61553_MOESM1_ESM.docx]

# Supplementary Information

# Bio-Based Protic Salts as Precursors for Sustainable Free-Standing Film Electrodes

**Alina Brzęczek-Szafran,^1^*, Magdalena Gwóźdź,^1^ Bartłomiej Gaida,^1^ Maciej Krzywiecki,^2^ Mirosława Pawlyta,^3^ Agata Blacha-Grzechnik,^1^ Anna Kolanowska,^1^ Anna Chrobok,^1^ Dawid Janas^1^***

^1^ Faculty of Chemistry,

Silesian University of Technology

44-100 Gliwice, Poland

alina.brzeczek-szafran@polsl.pl

dawid.janas@polsl.pl

^2^  Institute of Physics CSE, Department of Applied Physics

Silesian University of Technology

44-100 Gliwice, Poland

^3^ Faculty of Mechanical Engineering, Materials Research Laboratory

Silesian University of Technology


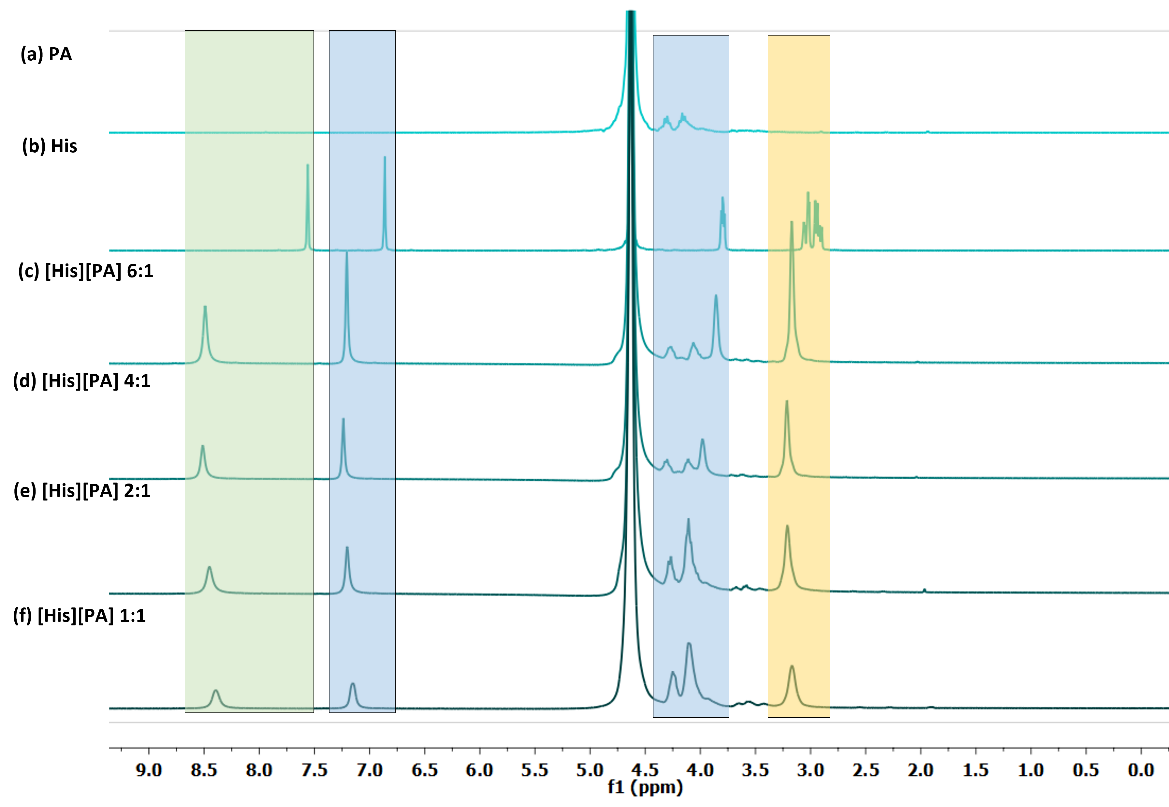


S1. ^1^H NMR spectra (400.112 MHz, 25 °C) of the systems based on phytic acid (PA) (a), histidine (His) (b), and their mixtures: [His][PA] 6:1 (c), [His][PA] 4:1 (d), [His][PA] 2:1 (e), [His][PA] 1:1 (f).


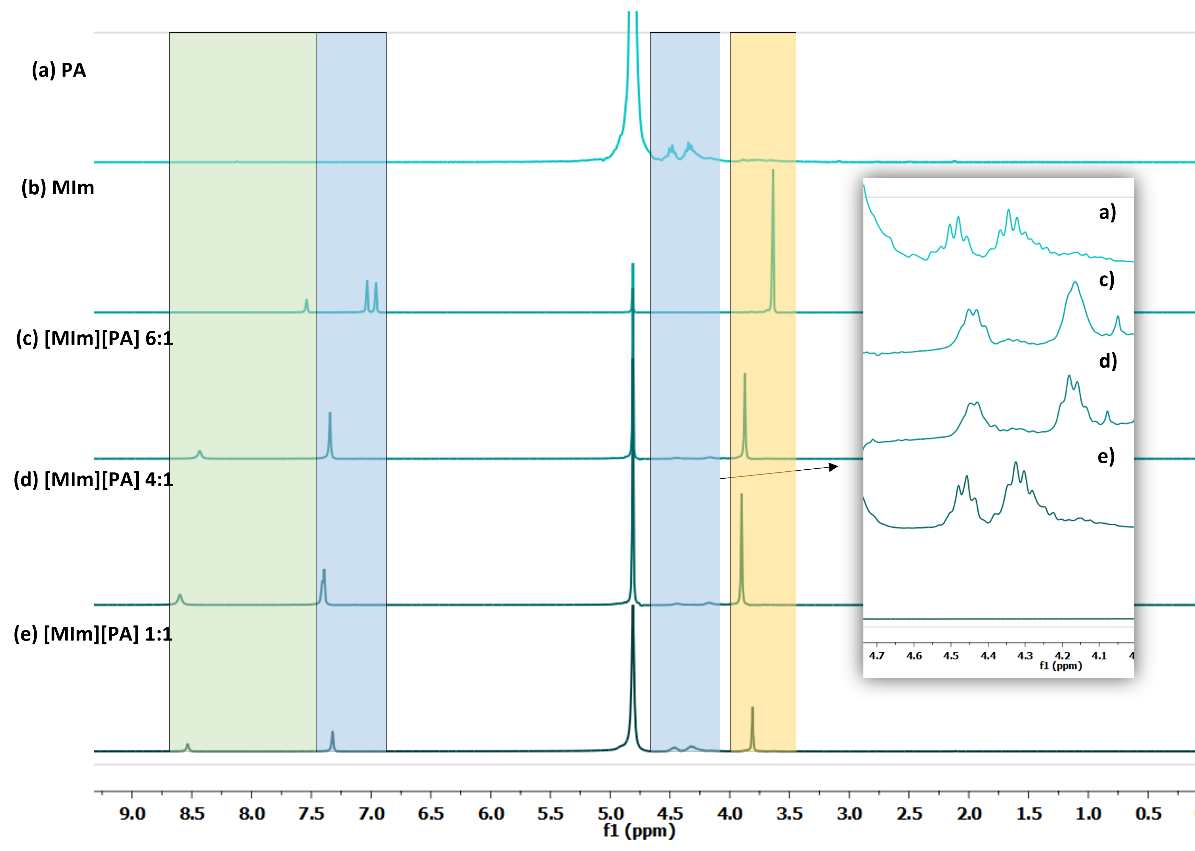


S2. ^1^H NMR spectra (400.112 MHz, 25 °C) of the systems based on phytic acid (PA) (a), 1-methylimidazole (MIm) (b), and their mixtures: [MIm][PA] 6:1 (c), [MIm][PA] 4:1 (d), [MIm][PA] 1:1 (e).


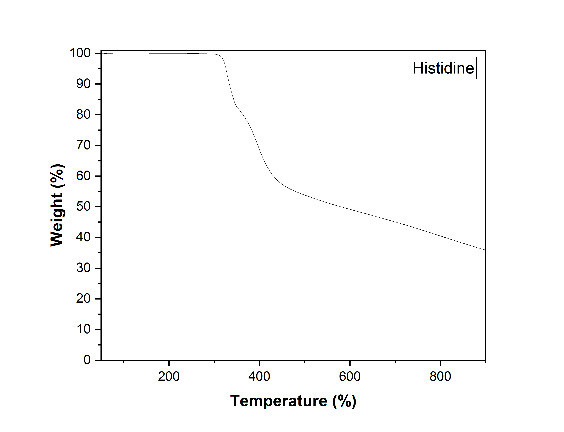


S3. Thermogravimetric analysis curve of histidine.


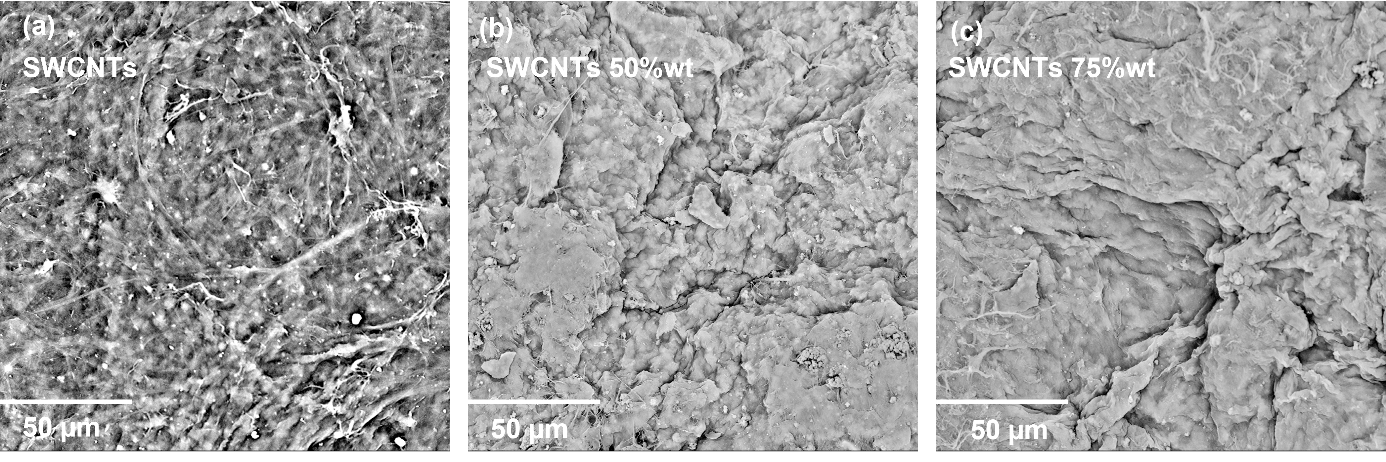


S4. SEM images of (a) pristine SWCNTs, (b) CM[MIm][PA] : SWCNTs 50:50 wt% composite, (c) CM[MIm][PA] : SWCNTs 25:75 wt% composite.
